# Functional Divergence and Emerging Roles of the ANO–TMC–TMEM63 Channel Families in Olfaction and Gustation

**DOI:** 10.3390/ijms27093989

**Published:** 2026-04-29

**Authors:** Hyungsup Kim

**Affiliations:** Research Institute of Pharmaceutical Sciences, College of Pharmacy, Sookmyung Women’s University, Seoul 04310, Republic of Korea; hkim@sookmyung.ac.kr

**Keywords:** anoctamin, TMC, TMEM63, chemosensation, olfaction, taste signaling, ion channels, sensory transduction

## Abstract

The olfactory and gustatory systems are essential for survival, enabling organisms to detect and respond to environmental chemical cues. Although canonical signaling pathways in smell and taste have been well defined, growing evidence highlights additional ion channel families as key modulators of sensory responses. Recent studies identify the anoctamin, transmembrane channel-like, and TMEM63 superfamily as a class of non-canonical sensory effectors that regulate signal amplification, excitability, and epithelial homeostasis across chemosensory systems. In the mammalian olfactory epithelium, specific anoctamin channels enhance odor-evoked responses and contribute to tissue homeostasis. In the gustatory system, salt detection is now understood to involve multiple parallel signaling pathways, with TMC4 emerging as a key contributor to high-salt and salt-associated taste sensing. These channel families are evolutionarily conserved across species, including *C. elegans*, *Drosophila*, and aquatic organisms, where they mediate chemosensation, mechanosensation, humidity detection, and osmoregulation. This functional versatility is supported by a shared structural architecture that enables selective ion conduction and, in some members, regulated phospholipid scrambling. This review proposes a unifying framework in which anoctamin and transmembrane channel-like proteins act as multimodal regulators of sensory signaling, linking environmental cues to cellular excitability and microenvironmental control and highlighting new principles of chemosensory organization and therapeutic potential.

## 1. Introduction

The olfactory and gustatory systems are evolutionarily conserved chemosensory modalities that translate external and internal chemical stimuli into neural signals via specialized receptors and ion channels. These senses are fundamental to survival, underpinning essential behaviors such as food detection, predator avoidance, and regulation of food intake. Their core organizational principles are preserved across species, ranging from invertebrates to humans.

The olfactory system represents a highly conserved sensory pathway across taxa, from mammals to invertebrates such as *Drosophila*. A defining feature of vertebrate olfaction is the compartmentalized localization of olfactory receptors (ORs), which are typically G protein-coupled receptors (GPCRs), to the cilia of olfactory sensory neurons (OSNs). These strategic positioning places ORs in direct contact with the external environment, enabling efficient detection of volatile odorants [1,2].

Odorant binding to ORs activates the olfactory-specific G protein (G_olf_), which in turn stimulates adenylyl cyclase III (ACIII). This enzymatic activation leads to elevated intracellular cAMP levels and triggers the opening of cyclic nucleotide-gated (CNG) cation channels. The resulting influx of Na^+^ and Ca^2+^ depolarizes OSNs and initiates action potential firing [3,4,5]. This primary depolarization is further amplified by Ca^2+^-activated chloride currents, which were originally characterized electrophysiologically in OSNs before their molecular identity was established [6,7]. Subsequent molecular studies identified ANO2 (also known as TMEM16B), a member of the anoctamin family, as the principal Ca^2+^-activated chloride channel mediating this secondary depolarizing amplification in olfactory sensory neurons [8].

In contrast to olfaction, the gustatory system is closely associated with food intake and nutrient evaluation and has traditionally been defined by five basic taste modalities: sweet, salty, sour, bitter, and umami. Sweet, bitter, and umami tastes are detected primarily by GPCRs expressed in Type II taste receptor cells (TRCs). These receptors converge on a shared intracellular signaling cascade involving phospholipase Cβ2 activation, intracellular Ca^2+^ release, and opening of the TRPM5 channel, culminating in membrane depolarization and neurotransmitter release [9,10].

Salty taste is mediated largely by the direct detection of sodium ions through epithelial sodium channels (ENaCs). Early physiological studies demonstrated that an amiloride-sensitive Na^+^ current underlies salt taste responses [11], and subsequent molecular and genetic analyses established ENaC as a principal component of this pathway, particularly for low-salt attraction [12,13].

The molecular identity of the sour taste transducer was elucidated more gradually and has historically been contentious. Initial studies implicated members of the polycystic kidney disease-like (PKD-like) transient receptor potential channel family [14,15,16]. However, genetic ablation of these channels produced only partial phenotypes, indicating that additional proton-sensing mechanisms must be involved [17]. Acid-sensing ion channels (ASICs) and hyperpolarization-activated cyclic nucleotide-gated (HCN) channels were also evaluated as principal sour transducers; however, available evidence does not support a primary role for these channels in sour taste transduction, although they may modulate proton sensitivity under specific conditions [18].

More recent work identified the proton-selective ion channel OTOP1 as the principal sour taste transducer in Type III TRCs. OTOP1 directly mediates proton influx response to acidic stimuli, leading to intracellular acidification, depolarization, and sustained action potential firing [19]. Functional studies demonstrated that *Otop1* knockout markedly disrupts intracellular acidification in Type III cells and substantially reduces gustatory nerve responses to acidic stimuli such as citric acid and hydrochloric acid [20].

Beyond the five classically defined taste modalities, accumulating evidence suggests that gustatory perception encompasses additional taste qualities and hybrid chemical-sensing mechanisms. In this expanded framework, OTOP1 has also been implicated in the detection of ammonium salts. Responses to NH_4_Cl in isolated Type III TRCs and gustatory nerves are substantially reduced or abolished in *Otop1*-deficient mice. Additionally, ammonium-induced aversive behavior is eliminated when *Otop1* deletion is combined with ablation of Type II taste cells, indicating a proton-channel-dependent component of ammonium salt taste [21].

Collectively, these findings support a model in which taste transduction relies on multiple TRC subtypes and partially overlapping signaling pathways, rather than strictly segregated receptor mechanisms. Consistent with this view, the chemical space accessible to taste has expanded to include additional stimuli such as fatty acids, peptides, and other nutrient-related cues, indicating a broader chemosensory capacity. These signals are processed by distinct TRC populations using diverse GPCRs and ion channels.

This evolving conceptual framework highlights the emergence of multifunctional ion channels and membrane proteins that participate in multiple sensory contexts and regulatory layers, extending beyond single-modality receptor functions. Accordingly, research attention has increasingly shifted from exclusively stimulus-specific receptors toward channel systems that also shape neuronal excitability, signal gain, and response dynamics. Notably, several channel families operate across multiple taste qualities and sensory conditions, supporting a more integrated view of chemosensory regulation.

Within this context, the Anoctamin/TMC superfamily is increasingly recognized as a shared ion channel platform that contributes to signal tuning and coordination across diverse sensory modalities. This review focuses on the expression patterns and functional roles of anoctamin family members in the olfactory and gustatory systems, with particular emphasis on their roles in excitability regulation, signal modulation, and sensory-related pathological responses. I also discuss emerging evidence for phospholipid scramblase activity within this family and its potential implications for chemosensory function.

## 2. ANO/TMC Superfamily Overview

The anoctamin (ANO) family, also known as the TMEM16 (transmembrane protein 16) family—a nomenclature originally based on its predicted protein structure rather than function—was established following the identification of ANO1 (TMEM16A) as a Ca^2+^-activated Cl^−^ channel in 2008 [22,23,24]. Subsequent studies demonstrated that the family comprises 10 members (ANO1–ANO10) and exhibits marked functional diversity, including both Ca^2+^-activated ion channel activity and Ca^2+^-dependent phospholipid scrambling. Within this framework, ANO1 and ANO2 function as classical Ca^2+^-activated Cl^−^ channels, whereas ANO6 (TMEM16F) and several related members operate predominantly as lipid scramblases, establishing two major functional branches within the family [25].

Sequence comparison analyses subsequently revealed that ANO proteins belong to a broader Transmembrane Channel/Scramblase (TCS) superfamily, which also includes TMC-like proteins and TMEM63/OSCA channels. The inclusion of these functionally distinct proteins within a common superfamily was unexpected based on physiological criteria alone. TMC1, for example, is a core component of the mechanotransduction apparatus in auditory hair cells [26,27,28], whereas TMEM63/OSCA proteins were initially identified as osmosensitive channels in plants [29,30]. This shared ancestry was first proposed through sequence-based bioinformatic analyses and later validated by structural studies showing similarity in molecular conformation [31,32,33].

High-resolution X-ray crystallography and cryo-electron microscopy structures of fungal nhTMEM16 and mouse Ano1 define a conserved anoctamin fold that serves as the structural core of this family [34,35]. Subsequent structural analyses demonstrated that both TMC proteins and TMEM63/OSCA channels adopt closely related architectural scaffolds, despite their divergent gating mechanisms and physiological roles [36,37,38,39,40].

Members of the TCS superfamily typically contain approximately 10 transmembrane helices per subunit and are often assembled as dimeric membrane complexes [37,41]. Despite this shared structural topology, gating and activation differ substantially. ANO1 retains conserved Ca^2+^-binding sites that support Ca^2+^-dependent gating, whereas TMC and TMEM63 proteins lack canonical Ca^2+^-coordinating residues and are associated with mechanosensory or osmosensory activation [37,42]. Additionally, TMEM63 channels have been proposed to function as monomers rather than dimers, highlighting further architectural and mechanistic divergence within a conserved structural framework [39].

Recent studies have highlighted the broad physiological diversity of the ANO family. In particular, the ANO family exhibits marked functional heterogeneity, combining variability in ion selectivity with phospholipid scramblase activity, which is an uncommon combination among ion channel proteins. Beyond ANO6, several members—including ANO3, ANO4, ANO7, and ANO9—have been reported to possess lipid scramblase activity [43,44,45]. Notably, ANO4 and ANO9 have also been described as cation-permeable channels rather than classical Ca^2+^-activated Cl^−^ channels, highlighting functional plasticity within the family [46,47]. Subcellular localization further distinguishes individual members: ANO8 and ANO10 are primarily associated with intracellular membranes, particularly the endoplasmic reticulum, and ANO10 is now recognized as an ER-resident lipid scramblase implicated in intracellular membrane homeostasis [48,49,50].

TMC proteins similarly exhibit pronounced subtype-specific functional variations. TMC1 and TMC2 are mechanically gated cation channels in inner ear hair cells that are essential for auditory mechanotransduction [27,51]. In addition to forming the ion-conducting pore, these proteins have been implicated in the regulation of phosphatidylserine (PS) at stereociliary membranes, suggesting a dual role in ion conduction and membrane lipid homeostasis [37]. Other TMC family members appear to serve distinct physiological functions. For example, TMC4 has been proposed to function as a voltage-dependent anion channel, based on emerging electrophysiological evidence [52], whereas TMC6 contributes to thermal sensing and calcium homeostasis [53,54].

Beyond this subtype specialization, ANO and TMC family members demonstrate broad functional versatility across tissues. This diversity is particularly evident within the sensory nervous system, where multiple studies indicate that these channels contribute to ionic signal modulation, response amplification, and local homeostatic regulation. Such roles are increasingly recognized in the olfactory and gustatory systems, where several members of the TCS superfamily function as modulators and amplifiers of receptor-driven ionic signaling pathways (Table 1).

## 3. Functions of Anoctamins in the Mammalian Olfactory System

**Figure 1:**
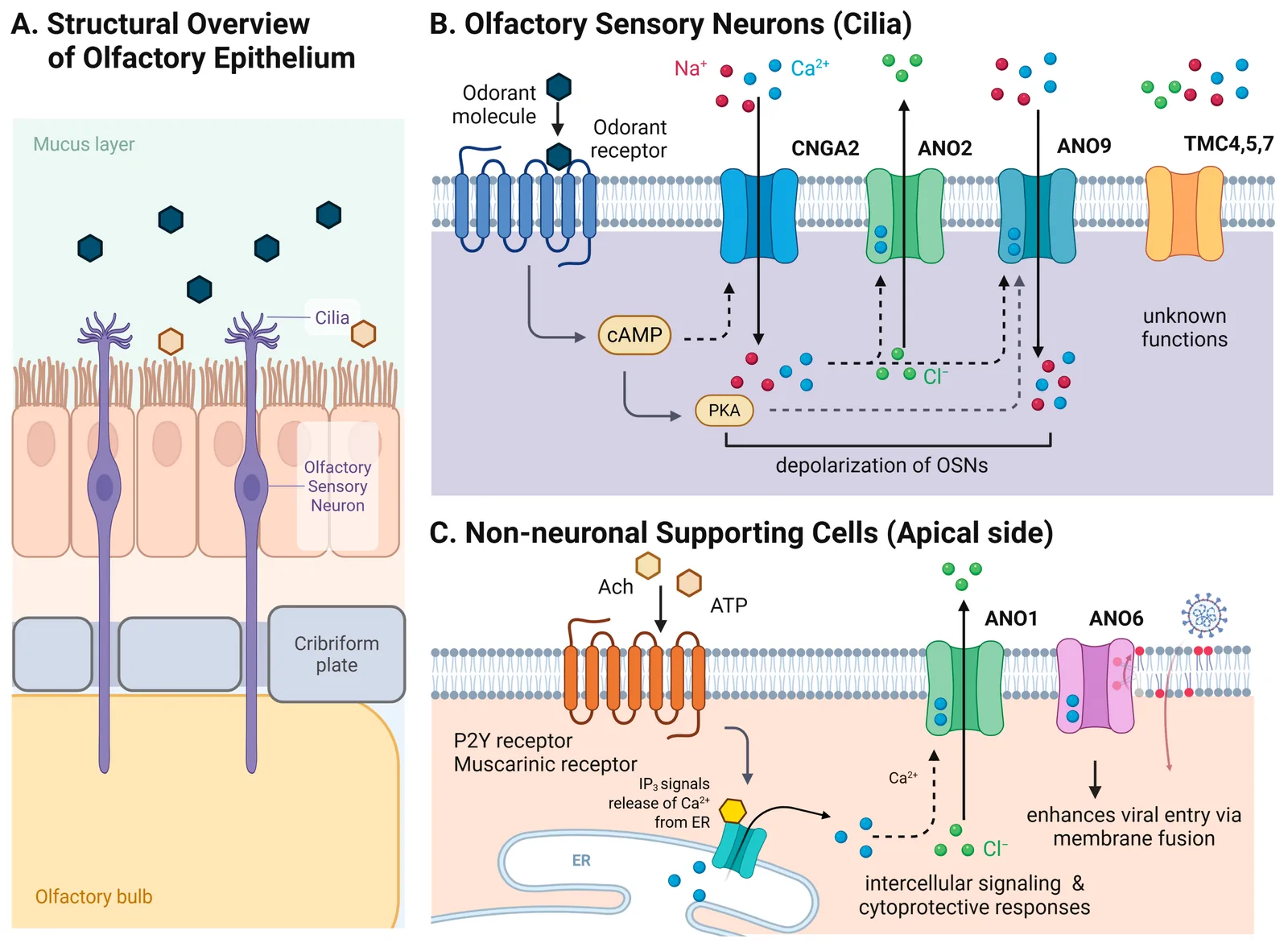
Roles of anoctamin and transmembrane channel-like protein channels in ion-channel amplification and regulation within the olfactory epithelium. (**A**) Structural organization of the olfactory epithelium, showing the mucus layer, sustentacular (supporting) cells, ciliated olfactory sensory neurons (OSNs), and their axonal projections through the cribriform plate to the olfactory bulb. Odorant molecules interact with receptors localized on the cilia of OSNs. (**B**) The primary olfactory transduction cascade in OSN cilia. Odorant binding activates the G protein–cAMP pathway, leading to the opening of cyclic nucleotide-gated channels and influx of Na^+^ and Ca^2+^. Elevated intracellular Ca^2+^ activates ANO2, while cAMP/PKA signaling may activate the cation channel ANO9. These channels act as auxiliary amplification mechanisms engaged downstream of the canonical signaling cascade. TMC4, TMC5, and TMC7 are expressed in OSNs, although their precise functions in olfactory signaling remain to be fully elucidated. (**C**) Nonneuronal supporting cells in the apical olfactory epithelium. Activation of purinergic (P2Y) and muscarinic receptors by extracellular ATP and acetylcholine triggers IP_3_-dependent Ca^2+^ release from the endoplasmic reticulum, leading to activation of ANO1. These pathways contribute to epithelial ionic regulation, intercellular signaling, and cytoprotective responses. ANO6 functions as a Ca^2+^-activated phospholipid scramblase and has been implicated in membrane remodeling and enhanced viral entry via membrane fusion. Created with BioRender.com (accessed on 19 March 2026).

Anoctamin family members play diverse and context-dependent roles in the mammalian olfactory system (Figure 1). Among anoctamin family members, ANO2 is the most extensively characterized channel in the mammalian main olfactory epithelium (MOE) and is established as the principal CaCC in OSN cilia. In this compartment, ANO2 mediates Ca^2+^-dependent Cl^−^ efflux following odorant-evoked Ca^2+^ entry through cyclic nucleotide-gated channels and contributes substantially to signal amplification [8,60].

Despite its dominant electrophysiological contribution, the functional indispensability of ANO2 remains debatable. Although *Ano2* knockout mice exhibit an almost complete loss of CaCC currents in OSNs, gross olfactory-driven behaviors are largely preserved [74]. Surprisingly, several studies have reported increased spike frequency or enhanced olfactory field potentials in the absence of ANO2, suggesting that ANO2-mediated Cl^−^ currents may exert a shunting or inhibitory influence under specific physiological conditions [75,76]. These observations indicate ANO2 double-edged functionality, involved in the maintenance of gross olfactory ability and fine regulation through its inhibitory function.

Nevertheless, *Ano2*-null mice exhibit behavioral impairments in detecting low-concentration odorants, including deficits in odor-guided foraging and tracking tasks [75,77,78]. These findings support a role for ANO2 in fine-tuning olfactory sensitivity near the perceptual threshold rather than mediating odor detection per se. Consistent with this interpretation, clinical studies of individuals homozygous for a large genomic deletion encompassing ANO2 revealed olfactory identification scores in the mild-to-moderate hyposmia range, without evidence of severe hyposmia or anosmia [79]. Olfactory performance did not differ significantly from that of heterozygous or wild-type family members, further supporting a contributory—rather than indispensable—role for ANO2 in human olfactory function [79].

More recently, ANO9 has emerged as an additional amplification channel in olfactory sensory neurons. In contrast to ANO2, which functions as a Ca^2+^-activated Cl^−^ channel, ANO9 operates as a cation-permeable channel that is activated downstream of odorant receptor signaling through the cAMP–protein kinase A (PKA) pathway. Electrophysiological recordings in cells coexpressing odorant receptors and ANO9 demonstrated that cAMP-dependent PKA activation gates ANO9 currents, establishing ANO9 as a downstream effector of canonical OR signaling [46,64]. Upon odorant stimulation, elevation of intracellular cAMP activates PKA, which in turn opens ANO9 channels, permitting additional Na^+^ and/or Ca^2+^ influx and thereby enhancing depolarization [64].

Consistent with this mechanism, *Ano9*-deficient mice exhibit reduced odor-evoked electrophysiological responses and impairments in odor-guided behavior, indicating that ANO9 contributes to normal olfactory sensitivity [64]. In chickens (*Gallus gallus*), reportedly, cAMP signaling fails to activate ANO9, an observation suggestive of divergence in amplification mechanisms across vertebrate lineages [64].

In contrast to ANO2 and ANO9, ANO1 is not expressed in olfactory sensory neurons; however, it is localized predominantly in non-neuronal cell types within the olfactory epithelium, including sustentacular and glandular cells of the Bowman’s glands and ducts [55,80,81]. ANO1 can be activated downstream of receptor pathways that elevate intracellular Ca^2+^ and mediates Ca^2+^-dependent Cl^−^ efflux in these supporting cells. For example, acetylcholine acting on M3 muscarinic receptors and ATP acting on P2Y purinergic receptors both trigger ANO1 and generate Ca^2+^-dependent Cl^−^ currents [55].

ANO1-mediated Cl^−^ currents are thought to contribute to epithelial membrane potential regulation, ionic homeostasis, and clearance of extracellular signaling molecules. Microvillar cells within the olfactory epithelium respond to high concentrations of odorants and toxic chemicals, acting as a local chemosensory surveillance system [81,82,83,84]. ANO1 appears to collaborate with the surveillance systems of microvillar cells for certain toxic substances, supported by the detection of severe epithelial damage in low-ANO1-expressing cells of the dorsal epithelial region [55,85,86]. Collectively, these observations support a potential protective role for ANO1 in maintaining epithelial integrity under chemical stress.

Taken together, these findings suggest that mammalian olfactory sensory neurons utilize multiple auxiliary amplification pathways that are recruited downstream of the core OR–cAMP–CNG transduction cascade. These include ANO2-mediated Ca^2+^-activated Cl^−^ currents and ANO9-mediated cation currents within OSNs, as well as ANO1-dependent regulatory functions in non-neuronal epithelial cells. This cell-type specific regulation underscores the diverse roles of anoctamin family members in olfactory processing and regulatory protection.

In addition to the MOE, the mouse nose contains a more specialized chemosensory organ, the vomeronasal organ (VNO). Vomeronasal sensory neurons (VSNs) are morphologically distinct from OSNs in the MOE and utilize different receptor repertoires and signal transduction cascades to detect pheromonal cues [74]. Notably, members of the ANO family are also expressed in the VNO. Both Ano1 and Ano2, which encode Ca^2+^-activated Cl^−^ channels, are localized to the apical microvillar region of the mouse VNO, where they co-localize with Trpc2, the primary ion channel mediating pheromone-evoked Ca^2+^ influx in VSNs [74,80,87]. This pattern contrasts with the MOE, where Ano1 and Ano2 are not co-expressed within the same cellular compartment. Consistent with this localization, Ca^2+^-activated Cl^−^ currents have been electrophysiologically recorded in VSNs. Inside-out patch-clamp recordings from dendritic knobs and microvilli confirmed that these currents are activated by intracellular Ca^2+^ [57]. Furthermore, selective deletion of Ano1 abolished Ca^2+^-activated Cl^−^ currents, indicating that ANO1 represents a major component of this conductance in the VNO [57,58]. In contrast, Ca^2+^-activated Cl^−^ currents remain detectable in *Ano2*-deficient mice, whereas loss of Ano1 markedly reduces these currents regardless of Ano2 expression, further supporting a dominant contribution of ANO1 to CaCC activity in VSNs [58]. Functionally, loss of Ano1 and Ano2 leads to reduced spontaneous and pheromone-evoked firing, as well as altered firing patterns in VSNs [58,59]. However, the absence of significant changes in VNO-dependent aggressive behavior indicates that these channels are not strictly essential for pheromone-driven behaviors but instead play modulatory roles in shaping sensory responses [58]. In addition, Ano9 expression has recently been reported in the VNO, although attributing its specific functional would require additional experimental evidence [64].

In contrast to anoctamin channels, the roles of TMC proteins in the mammalian olfactory system are still obscure. Nevertheless, transcriptomic analyses have detected several *Tmc* genes in OSNs. In particular, *Tmc4*, *Tmc5*, and *Tmc7* are selectively enriched in murine mature OSNs, suggesting that TMC proteins may serve neuron-intrinsic functions in olfactory signaling or homeostatic regulation, although their precise roles remain to be elucidated [73].

## 4. Functions in the Mammalian Gustatory System

**Figure 2:**
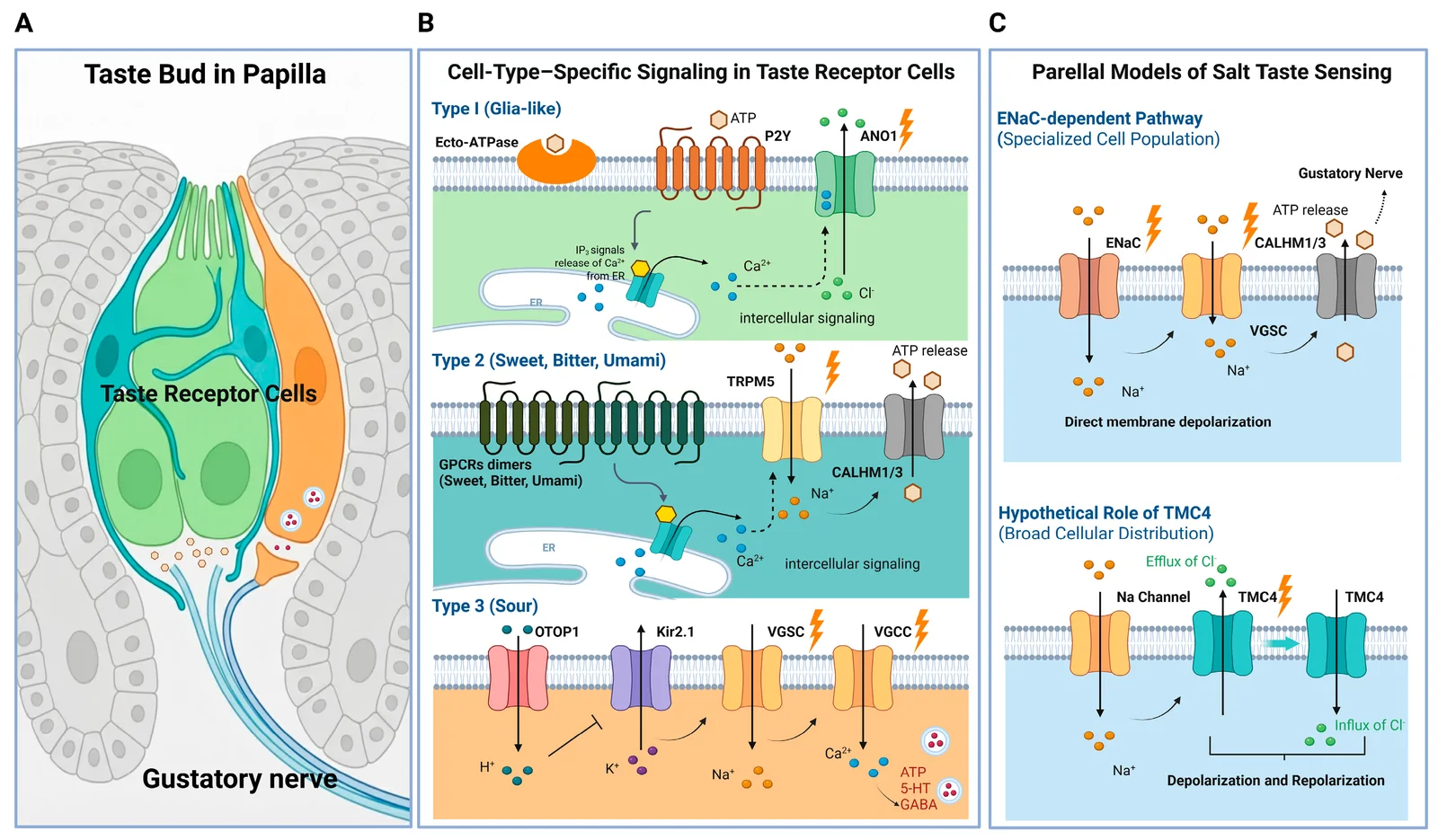
Cell-type-specific signaling pathways and models of salt taste sensing in mammalian taste buds. (**A**) Schematic representation of a taste bud within a lingual papilla, showing clustered taste receptor cells (TRCs) and their synaptic connections with gustatory nerve fibers. (**B**) Signaling mechanisms across major TRC subtypes. Type I (glia-like) cells contribute to ionic homeostasis and intercellular modulation. Extracellular ATP can activate purinergic receptors, triggering IP_3_-dependent Ca^2+^ release from the endoplasmic reticulum and subsequent activation of ANO1. Type II cells detect sweet, bitter, and umami tastants through GPCR dimers and a PLCβ2–IP_3_–Ca^2+^ cascade that activates TRPM5 channels, leading to depolarization and ATP release via CALHM1/3 channels. Type III cells mediate sour taste perception through the proton-selective channel OTOP1, with depolarization shaped by inhibition of Kir2.1 channels and activation of voltage-gated Na^+^ and Ca^2+^ channels (VGSC, VGCC), leading to vesicular neurotransmitter release. (**C**) Parallel models of salt-taste sensing. The established ENaC-dependent pathway mediates amiloride-sensitive low-salt detection through direct Na^+^ influx and membrane depolarization in a specialized cell population, followed by ATP release to gustatory nerves. In parallel, a proposed non-classical high-salt pathway involves broadly expressed channels, such as TMC4, which may contribute to depolarization–repolarization dynamics through Cl^−^ conductance and interactions with Na^+^ channel activity. This parallel-channel framework supports a multi-channel model for salty taste coding across TRC subtypes. Created with www.BioRender.com (accessed on 19 March 2026).

In mammals, the gustatory system detects a wide range of chemical stimuli through TRCs clustered within taste buds on the tongue. TRCs are classically categorized into three distinct types—Type I, Type II, and Type III—based on morphological features, molecular expression profiles, and functional characteristics (Figure 2). Each cell type contributes to taste perception through unique mechanisms of stimulus detection, signal amplification, and neurotransmission.

Type I cells are primarily glia-like support cells that maintain ionic homeostasis and regulate neurotransmitter clearance within the taste bud microenvironment [88]. Earlier studies suggested that Type I cells might participate directly in salt taste transduction because they express amiloride-sensitive epithelial Na^+^ channels [11,12,13]. More recent work, however, has revised this model, indicating that amiloride-sensitive salt taste is mediated by a distinct population of taste cells that express voltage-gated Na^+^ channels together with CALHM1/3 ATP-release channels [89]. These findings indicate that Type I cells appear to have collaborative regulatory role instead of being major salt detectors.

Type II cells lack conventional synapses with afferent gustatory fibers and instead signal via non-synaptic mechanisms. They detect sweet (TAS1R2–TAS1R3), umami (TAS1R1–TAS1R3), and bitter (TAS2R) compounds through GPCRs. Activation of these receptors engages a conserved cascade involving gustducin (GNAT3), PLCβ2, and IP_3_-mediated Ca^2+^ release [10,90]. The Ca^2+^ increase activates TRPM5 and TRPM4 channels, causing Na^+^ influx and depolarization, which triggers ATP release via CALHM1/3 to signal to afferent fibers [91,92].

In contrast, Type III cells form conventional synapses and use Ca^2+^-dependent neurotransmitter release, supported by SNAP25, GAD67, and voltage-gated Ca^2+^ channels [93,94,95,96,97]. Functionally, Type III cells are mainly responsible for detecting sour taste. These cells express OTOP1, an apical proton channel that mediates H^+^ influx and depolarization in response to acidic stimuli. This depolarization is further amplified by acid-mediated inhibition of the inwardly rectifying K^+^ channel KCNJ2 (Kir2.1), prolonging excitation and action potential firing [20,21,98].

In the classical model, TRCs are categorized into three cell types that mediate specific taste modalities via dedicated receptor-mediated mechanisms. Accumulating evidence, however, suggests that other ion channels may also contribute to taste signaling in a modality-specific manner. In this context, TMC4 has recently emerged as additional, new candidate involved in salt taste perception. Recent evidence suggests that TMC4 functions as a voltage-dependent Cl^−^ channel expressed in taste cells and is critical for detecting high concentrations of salt stimuli, including NaCl, KCl, and NH_4_Cl [52,65,66]. Electrophysiological analyses indicate that TMC4 is activated in depolarized taste cells following salt stimulation and that the resulting Cl^−^ conductance may influence repolarization kinetics and the duration of sensory signaling [52,65,66].

Beyond inorganic salts, TMC4 has also been implicated in the detection of salt taste peptides [89], providing a potential molecular mechanism for enhancing salt perception under low-sodium dietary conditions [68,69,70]. Consistent with a broader modulatory role, TMC4 is not confined to a single TRC subtype but is expressed across multiple taste cell subpopulations that co-express markers such as KCNQ1, gustducin, PLCβ2, and aromatic L-amino acid decarboxylase [71]. This expression profile suggests that TMC4 may function not only as a sensory transducer but also as a modulator of membrane excitability and signal tuning across taste cell types.

Behavioral analyses further support TMC4 in salt taste processing. In *Tmc4* knockout mice, amiloride-sensitive responses to low NaCl concentrations remain largely intact, whereas neural activation and avoidance behaviors elicited by high-salt stimuli are markedly reduced [52]. Moreover, *Tmc4*-deficient mice exhibit significantly diminished sensitivity to chloride-based salts (NaCl, KCl, NH_4_Cl), while responses to sodium gluconate—a non-chloride salt—remain relatively preserved [66]. These findings indicate a potential role for TMC4 in Cl^−^-dependent salt taste transduction. TMC4 has also been proposed to function as a negative regulator of the KCNQ1 (Kv7.1) potassium channel, potentially influencing the excitability and temporal dynamics of taste cell signaling [72], although this interaction requires further mechanistic validation.

In addition to TMC channels, anoctamin family members have been reported in taste buds. Both *Ano1* and *Ano2* transcripts have been detected in mouse taste cells, although their precise cell-type distribution remains controversial. Early single-cell transcriptomic analyses indicated predominant expression of *Ano1* in Type II cells and broader expression of *Ano2* across Type I and Type II cells [99]. However, subsequent immunohistochemical and functional studies failed to detect the Ano2 protein in mouse taste buds and instead identified Ano1 as the predominant anoctamin channel, with expression largely restricted to the apical microvilli of Type I supporting cells. Functional experiments further demonstrated that ANO1 can be activated downstream of purinergic receptor-dependent Ca^2+^ signaling [56]. During tastant stimulation, ATP released from Type II cells can diffuse to neighboring Type I cells and activate P2Y purinergic receptors, leading to intracellular Ca^2+^ elevation and subsequent activation of ANO1. The resulting Ca^2+^-dependent Cl^−^ currents are proposed to modulate membrane potential and influence intercellular signaling among taste cells [56]. Because Type I cells do not form classical synapses, ANO1-mediated Cl^−^ fluxes may participate in non-synaptic regulatory processes, including ionic microenvironment control, neurotransmitter clearance, and modulation of neighboring receptor cell activity.

Collectively, available data support the existence of multiple Cl^−^-dependent mechanisms in taste buds. TMC4 contributes to salt-evoked signaling, particularly at high stimulus intensities, whereas ANO1 functions primarily in epithelial and microenvironmental regulation. Most likely, these pathways provide modulatory support that complements the classical ENaC- and TRPM5-dependent taste transduction routes.

## 5. Evolutionary Conservation and Functional Diversity in Sensory Systems

**Figure 3:**
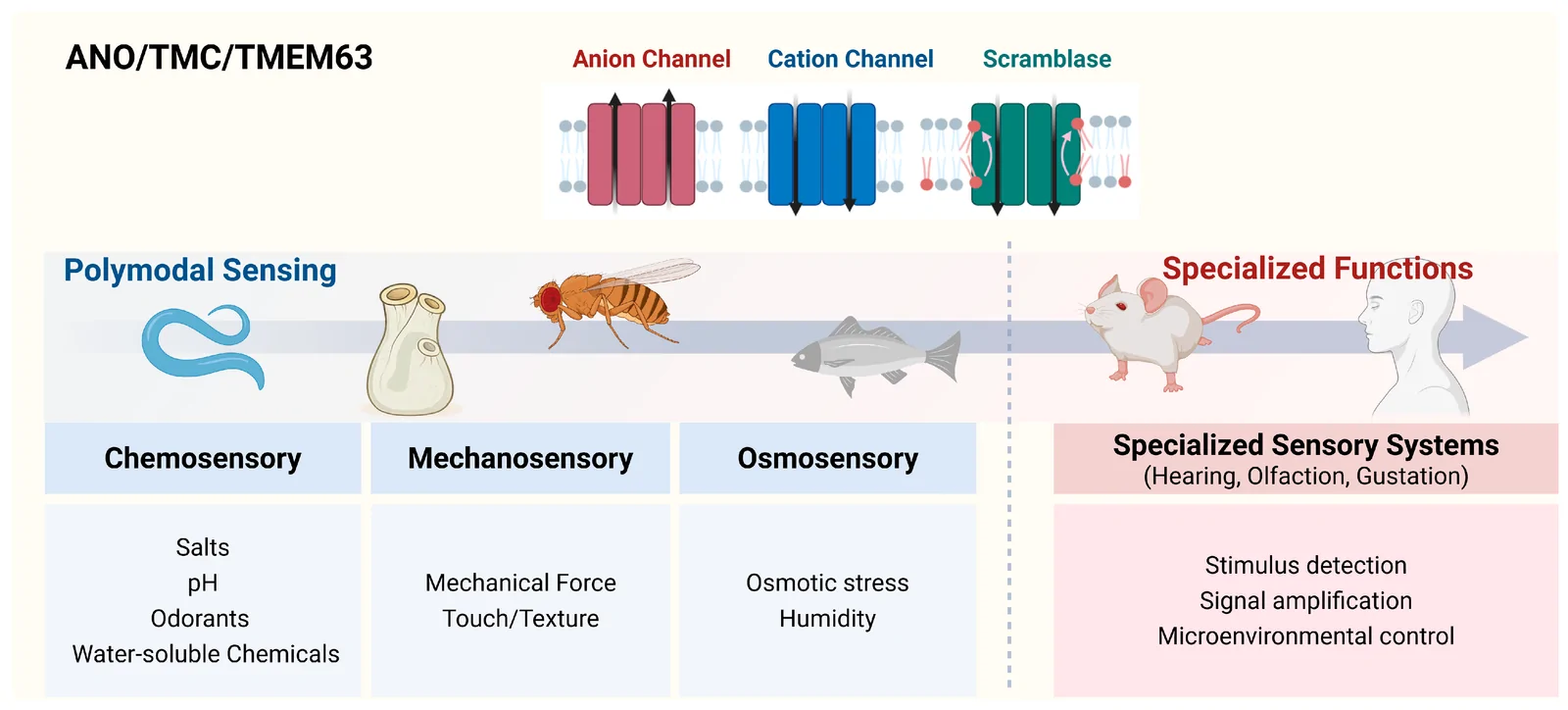
Evolutionary diversification of the ANO/TMC/TMEM63 channel superfamily in sensory systems.

In mammals, the roles of ANO/TMC superfamily members in olfactory and gustatory systems have been relatively well characterized, whereas accumulating evidence suggests that these channels function as polymodal sensory effectors across diverse biological contexts. Members of the ANO/TMC superfamily are conserved across a wide phylogenetic range, from protists and invertebrates to mammals, and are thought to represent ancient molecular solutions for converting environmental stimuli into electrical signals (Figure 3, Table 2). Recent work on genetically tractable model organisms, including *C. elegans* and *Drosophila melanogaster*, has suggested that these channels operate across multiple sensory modalities.

In lower organisms such as *Caenorhabditis elegans*, olfactory and gustatory modalities are not distinctly segregated; rather, they function as an integrated chemosensory system. For example, AWA, AWB, and AWC neurons primarily respond to volatile compounds and perform olfaction-like roles, whereas ASE, ASH, ASI, and ASG neurons respond to water-soluble chemicals, ion concentrations, and osmotic pressure, resembling gustatory functions [100,101]. Unlike mammals, *C. elegans* lacks canonical OR genes; instead, it expresses a wide array of GPCR family receptors and ion channels capable of directly detecting various environmental cues such as pH, osmolarity, ionic gradients, and mechanical force [102]. Such organization blurs the classical distinction between olfaction and taste, highlighting the importance of amplification mechanisms, signal integration, and precise regulation of the sensory microenvironment in chemosensory processing.

The *C. elegans* genome encodes multiple members of both the TMC and anoctamin families, including *tmc-1*, *tmc-2*, *ano-1*, and *ano-2*. These channels exhibit both conserved and divergent activation mechanisms relative to their mammalian counterparts, suggesting a structurally related yet functionally versatile gating framework. Recent high-resolution structural analyses of TMC-1 and TMC-2 have revealed architectures consistent with mechanosensory gating, supporting the idea that mechanotransduction represents an evolutionarily conserved function of the TMC family [28,38,40]. Beyond mechanosensation, TMC-1 functions in salt sensing [103]. It is expressed in polymodal ASH avoidance neurons and is required for neuronal activation and avoidance behavior in response to high NaCl concentrations). Importantly, TMC-1 is dispensable for ASH-mediated responses to other aversive stimuli, such as hyperosmotic stress or certain chemical cues, suggesting stimulus specificity for Na^+^ sensing [103]. Subsequent studies further implicated TMC-1 in alkaline avoidance, where channel activation is associated with inward currents in nociceptive sensory neurons [104].

Anoctamin family members in *C. elegans* also contribute to sensory signaling, although through distinct mechanisms. The *ano-1* gene encodes a Cl^−^ channel involved in mechanosensation through a calcium- and integrin-binding protein-dependent pathway [105]. Activation of ANO-1 mediates behavioral responses to mechanical stimuli, suggesting that anoctamin channels participate in sensory modalities beyond classical Ca^2+^-activated Cl^−^ conductance observed in mammals [105]. Together, these findings suggest that TMC and anoctamin channels may function in parallel or complementary sensory pathways, with activation mechanisms tailored to specific stimulus modalities and cellular contexts.

In the basal chordate *Ciona intestinalis*, Ano5 and Ano6 have been implicated in detecting environmental cues that regulate larval settlement and metamorphosis [106]. These channels are expressed in adhesive papillae—specialized sensory structures responsible for substrate recognition. They also mediate intracellular Ca^2+^ influx in response to both mechanical contact with rigid surfaces and chemical stimulation, such as exposure to 10 mM ammonium chloride, both of which are known to trigger metamorphic onset. Knockdown of *Ano5* and *Ano6* significantly diminishes Ca^2+^ signaling and impairs metamorphic initiation, suggesting a critical role for these channels in integrating external cues during early developmental transitions [106].

In teleost fish, ciliated olfactory receptor neurons are present and utilize a cAMP-dependent signaling pathway in response to odorant stimulation [107,108]. In addition, studies in rainbow trout have shown that amino acid-evoked inward currents are partly mediated by Ca^2+^-activated Cl^−^ conductance, suggesting that Cl^−^-dependent signal amplification mechanisms are functionally conserved in fish olfactory neurons [108]. Apart from sensory transduction, anoctamin channels have also been implicated in osmoregulatory processes in aquatic vertebrates. Teleost fish, such as zebrafish and other species, must continuously regulate ion transport to adapt to changes in environmental salinity during transitions between freshwater and seawater [109]. In the euryhaline fish *Fundulus heteroclitus*, which adapts to fluctuating salinity environments, Ano1 expression is upregulated in gill epithelial cells under high-salinity conditions, supporting a role in chloride-dependent osmotic regulation [110]. Similarly, the functional properties of zebrafish Ano1a imply that it may also facilitate chloride efflux, potentially serving a comparable role in osmoregulatory homeostasis [111]. These findings underscore the conserved role of anoctamin family members in responding to osmotic challenges across chordate lineages.

In amphibians, Ca^2+^-activated Cl^−^ conductance has been directly demonstrated in the cilia of frog OSNs using patch-clamp recordings [6]. In contrast, electrophysiological and functional studies of olfactory transduction in reptiles remain limited; however, in species such as snakes, both the main olfactory system and the accessory olfactory system are well developed and are mediated by OR and V1R/V2R receptors, respectively. Transcriptomic analyses have revealed the expression of key GPCR–cAMP signaling components, including *Adcy3*, suggesting that the fundamental olfactory transduction cascade appears to be conserved [112]. Nevertheless, the expression and functional roles of anoctamin family channels in reptiles remain largely unknown.

In contrast, although birds are generally considered to lack a functional VNO [113], recent studies have demonstrated that *Or*/*Taar*, *Gnal*, *Adcy3*, and *Cng* channel subunits, along with *Ano2* mRNA, are expressed in the olfactory epithelium of *Gallus gallus* at the qPCR level. These findings suggest that the canonical olfactory transduction cascade is conserved in birds [114]. Interestingly, ANO9 cloned from the *Gallus gallus* was reported to lack functional activity in electrophysiological assays, implying potential functional divergence of anoctamin family members or presence of alternative signaling mechanisms in the avian olfactory system [64].

In addition to vertebrate taste systems, TMC and TMEM63 family channels have been repurposed in insects to support mechanosensory and hygrosensory behaviors. In *Drosophila*, TMC is expressed in multidendritic (md-L) neurons located at the base of taste hairs and is required for sensing food texture, including hardness and viscosity [115]. These neurons modulate feeding behavior in a graded manner, depending on the direction and intensity of mechanical stimulation. Loss of TMC impairs discrimination of food particle size and viscosity, resulting in altered feeding preferences. Humidity sensing in *Drosophila* is essential for selecting favorable microenvironments for survival and reproduction, and is mediated by Or42b-expressing olfactory sensory neurons. In these cells, ambient humidity induces mechanical deformation of cuticular structures [116]. This mechanotransduction process requires TMEM63 channel activity; flies lacking *tmem63* exhibit abnormal humidity preference behaviors that can be rescued by expression of human TMEM63B, indicating functional conservation across species [116].

Consistent with this evolutionary conservation, TMEM63B also functions in mammalian osmosensory circuits. TMEM63B is expressed in neurons of the subfornical organ, where it functions as a mechanosensitive ion channel that detects cellular shrinkage under hyperosmotic conditions and contributes to dehydration sensing [117,118]. Mice lacking *tmem63b* show reduced neuronal responses to hyperosmotic stimulation and decreased water intake, supporting a conserved role for TMEM63B channels in osmomechanosensory signaling. Collectively, these findings illustrate how the TMEM63/OSCA channel family—originally identified as plant osmosensors—has been functionally repurposed to support humidity and osmolarity sensing in animals [116].

Anoctamin-mediated Ca^2+^-activated Cl^−^ conductance has been observed across multiple vertebrate species and is thought to contribute to olfactory signal amplification in fish, amphibians, birds, and mammals. Along with this established function, members of the ANO/TMC channel superfamily have been repeatedly adapted across evolutionary scales to serve as molecular effectors for sensing and regulating diverse environmental and physiological cues, including ionic composition, pH, texture, humidity, and osmolarity. These functions are critical for survival, homeostasis, and behavioral adaptation across species.

Anoctamin (ANO), transmembrane channel-like (TMC), and TMEM63 proteins share conserved multi-pass membrane architectures but have diversified to support distinct sensory functions. Across species, these channels contribute to polymodal environmental sensing, including chemo-, mechano-, and osmosensation. In mammals, further specialization has emerged in sensory systems such as hearing, olfaction, and gustation, where these channels participate in stimulus detection, signal amplification, and regulation of the local sensory microenvironment. The above figure was created with www.BioRender.com (accessed on 19 March 2026).

**Table 2:** Polymodal Sensory Roles of ANO/TMC Family Genes Across Species.

Gene	Species	Expression Site/Tissue	Sensory Function	References
*ano-1*	*Caenorhabditis elegans*	Mechanosensitive neurons	CaCC and Mechanosensory (CIB-dependent activation)	[105]
*ano-2*	*Caenorhabditis elegans*	*C. elegans* neurons	Putative CaCC, function unresolved	
*A* *no* *1*	*Fundulus heteroclitus*	Gill epithelium (salinity-dependent expression)	Osmoregulation via Cl^−^ transport	[110]
*A* *no* *1a*	*Danio rerio*	Unspecified	Putative Cl^−^ channel; potential role in osmoregulation	[111]
*Ano5*, *Ano6*	*Ciona intestinalis*	Adhesive papillae	Metamorphosis and environmental cue sensing	[106]
*tmem63*	*Drosophila melanogaster*	Or42b+ olfactory neurons	Humidity sensing (cuticle deformation transduction)	[116]
*Tmem63b*	*Mus musculus*	SFO neurons	Thirst/osmosensation (cell shrinkage detection)	[117,118]
*tmc*	*Drosophila melanogaster*	md-L neurons (taste hairs)	Texture detection (hardness, viscosity)	[115]
*tmc-1*	*Caenorhabditis elegans*	ASH polymodal neurons	Mechanosensation, salt chemosensation, alkaline avoidance	[38,103,104]
*tmc-2*	*Caenorhabditis elegans*	*C. elegans* neurons	Presumed mechanosensory function	[40]

## 6. Scramblase Activity and Sensory Implications

Members of the anoctamin family, most prominently ANO6, act as Ca^2+^-activated phospholipid scramblases that catalyze the bidirectional translocation of PS across the plasma membrane [119]. While PS exposure is classically associated with apoptosis or cellular damage, it is increasingly recognized as a regulated physiological process involved in synaptic remodeling, cell–cell communication, and tissue organization. Dynamic modulation of membrane lipid asymmetry may therefore influence functional regulation of sensory cells.

Although direct experimental evidence in the sensory system remains limited, early biophysical studies have suggested that membrane lipid composition may influence sensory neuron function. Changes in phospholipid content were shown to alter electrical responses to chemical stimuli [120], and exogenous application of PS enhanced olfactory sensitivity by lowering detection thresholds, albeit under non-physiological conditions [121].

Disruption of phospholipid asymmetry and PS externalization has since been linked to dysfunction in various model systems. In *Drosophila*, loss of the phospholipid flippase dATP8B reduces olfactory sensitivity in OR-expressing neurons [122]. Moreover, abnormal PS exposure resulting from flippase deletion or scramblase overexpression induces dendritic degeneration during larval stages and axonal loss in adult olfactory circuits, ultimately impairing sensory function [123]. In mammals, the scramblase Xkr8 promotes PS exposure during the developmental axon pruning, facilitating elimination of inappropriate synaptic connections [124]. Together, these studies suggest that maintenance of phospholipid asymmetry represents a conserved mechanism for sensory neuron stability and circuit refinement.

In addition to these roles, emerging evidence suggests that scramblase-mediated lipid remodeling may also be involved in broader pathological contexts. One clinically relevant example is virus-associated cellular pathology. SARS-CoV-2 preferentially infects sustentacular cells in the olfactory epithelium that coexpress ACE2 and TMPRSS2 [125]. These supporting cells express relatively high levels of ANO6, and viral spike protein engagement has been associated with increased ANO6 scramblase activity [61]. Activated ANO6 disrupts membrane lipid asymmetry and promotes PS externalization, which can facilitate membrane fusion between neighboring cells. This process contributes to the formation of multinucleated syncytia and disruption of epithelial architecture [62,63]. In addition, the ANO6 scramblase inhibitor niclosamide has been reported to suppress SARS-CoV-2 spike-induced cell fusion, further supporting the functional relevance of this pathway [63].

Collectively, these findings suggest that regulated scramblase activity and membrane lipid remodeling play important roles in maintaining sensory cell structure and microenvironment organization. While their roles in physiological contexts are well supported, dysregulation of these processes may also influence cellular vulnerability and tissue integrity under pathological conditions.

## 7. Future Perspectives

The ANO/TMC superfamily of ion channels has emerged as a functionally versatile group of membrane proteins operating across multiple sensory modalities, including olfaction and taste, in a wide range of species. Through distinct gating mechanisms and dual functional capacities—ion conduction and Ca^2+^-dependent phospholipid scrambling—these channels contribute to the efficiency and precision with which sensory cells transduce external stimuli into electrical signals. Their strong evolutionary conservation, spanning from *C. elegans* to mammals, suggests that ANO/TMC proteins represent a conserved molecular toolkit that organisms have repeatedly exploited to adapt to diverse environmental cues.

Growing evidence indicates that ANO/TMC channels may also represent promising therapeutic targets in sensory disorders. In the olfactory system, genetic variation and dysregulated expression of signal-modulating ion channels have been associated with reduced odor sensitivity and impaired sensory performance. In parallel, channels with scramblase activity have been implicated in virus-associated epithelial pathology, particularly in the context of infection-induced disruption of membrane lipid asymmetry. These observations raise the possibility that targeting scramblase-associated channel activity could represent a rational therapeutic strategy for nasal epithelial infections.

In the gustatory system, high-salt taste perception is now recognized to arise from multiple parallel signaling pathways rather than a single, receptor-defined mechanism. Ion channels contributing to this non-classical high-salt signaling axis, therefore, represent attractive candidates for modulating salt taste intensity. Selective tuning of these pathways, including modulation of channels such as TMC4, may offer novel approaches for salt reduction or salt substitution strategies aimed at improving dietary compliance without compromising palatability.

Future studies are needed to elucidate how ANO/TMC channels are differentially regulated across sensory cell subtypes, how they interact with other ion channels, and how local membrane lipid environments modulate their functional output. Collectively, the ANO/TMC superfamily constitutes a key molecular platform for stimulus detection and signal transduction in sensory cells and a shared ion channel platform involved in diverse sensory and homeostatic signaling processes, with growing implications for both basic sensory biology and the development of translational interventions.

## Figures and Tables

**Table 1:** Chemosensory Roles of ANO/TMC Family Genes in Mammalian Olfaction and Gustation.

Protein	Expression Site/Tissue	Functions	References
Ano1	Olfactory supporting (sustentacular) cells	Cl^−^-dependent ionic environment modulation, ATP response	[55]
	Taste receptor Type I cells	Cl^−^-dependent ionic environment modulation	[56]
	Vomeronasal sensory neurons	Modulation of pheromone-evoked responses and firing patterns	[57,58,59]
Ano2	Olfactory neuron cilia	Olfactory signal amplification	[8,60]
	Vomeronasal sensory neurons	Modulation of pheromone-evoked responses and firing patterns	[58,59]
Ano6	Sustentacular cells	Phospholipid scramblase activity; involvement in SARS-CoV-2-related syncytia formation	[61,62,63]
Ano9	Olfactory neuron cilia	Olfactory signal amplification	[64]
	Vomeronasal organ	Unknown	[64]
Tmc4	Taste receptor cells (multiple TRC subtypes)	Cl^−^-dependent high-salt taste detection; modulation of KCNQ1 channel activity	[52,65,66,67,68,69,70,71,72]
	Olfactory sensory neurons (mRNA)	Unknown; transcript expression reported	[73]
Tmc5	Olfactory sensory neurons (mRNA)	Unknown; transcript expression reported	[73]
Tmc7	Olfactory sensory neurons (mRNA)	Unknown; transcript expression reported	[73]

## Data Availability

No new data were created or analyzed in this study. Data sharing is not applicable to this article.
